# Editorial: Artificial intelligence for mental disorder prevention and diagnosis: Technologies and challenges

**DOI:** 10.3389/fpsyt.2023.1161158

**Published:** 2023-03-30

**Authors:** Peiying Zhang, Neeraj Kumar, Muhammad Zakarya, Laith Abualigah

**Affiliations:** ^1^Qingdao Institute of Software, College of Computer Science and Technology, China University of Petroleum (East China), Qingdao, China; ^2^State Key Laboratory of Integrated Services Networks, Xidian University, Xi'an, China; ^3^Department of Computer Science and Engineering, Thapar Institute of Engineering and Technology, Patiala, India; ^4^School of Computer Science, University of Petroleum and Energy Studies, Dehradun, Uttarakhand, India; ^5^Faculty of Computing and Information Technology, Sohar University, Sohar, Oman; ^6^Department of Computer Science, Abdul Wali Khan University, Mardan, Pakistan; ^7^Faculty of Information Technology, Middle East University, Amman, Jordan

**Keywords:** artificial intelligence, diagnosis, adjunctive treatment, model, mental disorders

## 1. Introduction

Artificial intelligence (AI) has made an indelible contribution to the progress and development of human society, which has major applications in the fields of national defense, transportation, finance, logistics, education, etc. As a branch of computer science, AI has made significant breakthroughs in machine translation, intelligent control, language and image processing, automatic programming, and storage computing. Artificial intelligence-driven healthcare technologies are rapidly evolving into solutions that are applicable to clinical practice. AI has greatly helped improve health outcomes by using patient-relevant clinical data for early diagnosis of symptoms and better prognosis. Therefore, there is reason to believe that AI can make greater contributions to the prevention and diagnosis of mental disorders.

There have been relevant representative studies based on AI technology that have made breakthrough contributions in the prevention, diagnosis, and treatment of other diseases. In order to improve the applicability of AI technology in the mental disorder field, it is necessary to propose new theories, technologies, architectures, algorithms, and mechanisms. The goal of this Research Topic is to bring together relevant researchers from industry and academia to share their latest findings and developments in the application of AI in medicine, especially in the prevention and diagnosis of mental disorders. It was our unexpected pleasure to have 4 papers in this topic which reported new exciting findings and cutting edge methodologies.

## 2. Research overview

In this Research Topic, four articles focus on the performance of artificial intelligence in the field of mental disorders, such as symptom diagnosis, adjunctive treatment, and model optimization.

**Depression recognition model:** Xie et al. explored the application of the depression recognition model (DR model) based on AI machine learning (ML) in the diagnosis of depression. They explored the rhythms of adrenocorticotropic hormone (ACTH) and cortisol in depressed and anxious patients and their effects on mental status, and extracted data features based on electroencephalo-graph (EEG) data from different patients to construct a DR model for diagnosis (Xie et al.).

**AI-based chatbot micro-intervention for parents:** Entenberg et al. investigated the effectiveness of intelligent chatbots to help parents with parenting. The authors recruited a sample of 170 parents with at least one child to participate in the chatbot intervention. Parents can learn skills for parenting such as effective praise techniques. This study analyzed the effectiveness of parenting chatbot micro-interventions, the learning gained by participants from pre to post-intervention, and the user characteristics of parents. According to the experimental results, mental health chatbots are a promising method for busy parents of children and adolescents to enhance parenting skills (Entenberg et al.).

**Point of interest (POI) recommendation based on preference perception:** Graph embeddings and graph neural networks are fail to capture deep graph structure information in point-of-interest recommendation tasks, resulting in unsatisfactory representation results. To address the above issues, Shu et al. proposed a general framework based on the diffusion of preference-aware graphs to capture the deep structural information neglected in most existing methods for POI recommendation.

**Understanding psychiatric illness through natural language processing:** The diagnosis of mental disorders is mainly based on the level of experience of each psychiatrist and often lacks objectivity. To this end, Kishimoto et al. created a large Japanese speech dataset, used natural language processing to quantify the linguistic features of mental disorders and neurocognitive disorders. Finally, objective and easy-to-use biomarkers were developed. These biomarkers quantify the linguistic features of mental disorders, allow more detailed examination of their correlation with other clinical indicators, and improve the accuracy of disease prediction (Kishimoto et al.).

## 3. Conclusion

These typical research efforts are artificial intelligence for the adjunctive treatment of psychiatric conditions, diagnosis and optimization of neural network models, which are important for the development of novel network models and intelligent diagnostic techniques. The development of artificial intelligence provides new ways of diagnosis and treatment in medicine. We look forward to closer interaction as well as breakthroughs between AI and the field of mental disorders. Finally, we thank all contributing authors for their novel research works and publishers, as well as the editorial team for their efforts in organizing this Research Topic.

## Author contributions

All authors listed have made a substantial, direct, and intellectual contribution to the work and approved it for publication.

